# Observational Study of the Clinical Characteristics and Short-Term Outcomes of Kidney Transplant Recipients Diagnosed With COVID-19 Infection (SARS-CoV-2) Requiring Hospitalization in New Orleans

**DOI:** 10.31486/toj.21.0008

**Published:** 2021

**Authors:** Sixto Giusti, Shai Chazin, Pradeep Vaitla, Kofi Atiemo, Mohammad Atari, Anil Paramesh, Hoonbae Jeon, Aldo Torres-Ortiz, Ravi Thimmisetty, Jorge Garces

**Affiliations:** ^1^ Department of Medicine, Section of Nephrology and Hypertension, Tulane University School of Medicine, New Orleans, LA; ^2^ Department of Surgery, Kidney and Pancreas Transplant Program, Tulane University School of Medicine, New Orleans, LA; ^3^ Department of Medicine, Section of Nephrology and Transplant, University of Mississippi Medical Center, Jackson, MS; ^4^ Department of Internal Medicine, Ochsner Clinic Foundation, New Orleans, LA; ^5^ Multi-Organ Transplant Institute, Ochsner Clinic Foundation, New Orleans, LA; ^6^ The University of Queensland Faculty of Medicine, Ochsner Clinic Foundation, New Orleans, LA

**Keywords:** *Acute kidney injury*, *COVID-19*, *immunosuppression*, *mortality*, *transplant recipients*

## Abstract

**Background:** Kidney transplant recipients are at increased risk of severe disease and death caused by coronavirus disease 2019 (COVID-19) infection. The role of immunosuppressive medications in the clinical presentation, disease course, and outcomes is not well understood.

**Methods:** We analyzed kidney transplant recipients diagnosed with COVID-19 and requiring hospitalization during the initial infection surge at 2 large transplant centers in New Orleans, Louisiana, between February 1, 2020 and April 30, 2020. Patient presentation, clinical course, kidney transplant function, and postdischarge details are included in this analysis.

**Results:** Twenty-three kidney transplant recipients hospitalized with COVID-19 were included in the study. The majority of patients were Black (95.7%). Diabetes, hypertension, and obesity were present in more than 50% of the patients. The most common presenting symptom was fever, present in 52.2% of patients. All patients were managed with reduction in immunosuppression. Patients received azithromycin (60.9%), hydroxychloroquine (47.8%), remdesivir (8.7%), and intravenous methylprednisolone pulse (8.7%). The average length of stay was 4.5 days (range, 2-18 days). In this study population, 73.9% of the patients sustained acute kidney injury, with an average peak serum creatinine of 3.81 mg/dL. Twenty-six percent of the patients required renal replacement therapy. Seventy-seven percent of patients developed proteinuria (at least 1+ proteinuria on urinalysis). Of the patients in this population who required mechanical ventilation (39.1%), 77.8% died. Overall, 30.4% of patients died of COVID-19–related complications during admission. Of the 16 patients discharged, the average serum creatinine at discharge was 2.09 mg/dL compared with an average preadmission serum creatinine of 1.8 mg/dL.

**Conclusion:** During the initial COVID-19 infection surge in New Orleans, we noted that kidney transplant recipients had initial symptoms similar to the general population. However, we recorded a higher incidence of acute kidney injury and death compared to nontransplant patients. Patients who required mechanical ventilation had a high mortality rate. Black patients are overrepresented in our study.

## INTRODUCTION

According to data from the Centers for Disease Control and Prevention (CDC), the number of coronavirus disease 2019 (COVID-19) cases in the United States had reached 8.68 million as of October 27, 2020. The overall case fatality rate was 3%, with in-hospital mortality of 14.2%.^1,2^ Cases of transplant patients infected with severe acute respiratory syndrome coronavirus 2 (SARS-CoV-2), the virus that causes COVID-19, have reported mortality rates ranging from 18% to 32%.^3-7^ The increased mortality is likely related to the comorbidities hypertension, diabetes, and chronic kidney disease (CKD) among transplant recipients, which are associated with higher rates of mortality among patients with COVID-19 infection.^4,8^ Furthermore, Black patients are at higher risk for severe COVID-19 infection and death compared to White patients.^9,10^

The role immunosuppression plays in disease presentation, clinical course, and outcomes is currently under investigation. Reports suggest that reducing immunosuppressive medications in COVID-19–infected transplant recipients is indicated, particularly antiproliferative agents and/or calcineurin inhibitors.^3-7,11-15^ Additionally, atypical presentations among transplant recipients, such as being less likely to present with fever, possibly reflect immunologic changes in patients taking immunosuppressive medications.^16^

We report the clinical presentations and short-term outcomes of COVID-19–infected kidney transplant recipients during the initial COVID-19 infection surge in New Orleans, Louisiana.

## METHODS

We analyzed COVID-19–infected kidney transplant recipients admitted at 2 transplant centers in New Orleans between February 1, 2020 and April 30, 2020. Inclusion criteria were kidney transplant recipients with a functioning allograft who were >18 years and who demonstrated polymerase chain reaction positivity for SARS-CoV-2 infection. Patients with incomplete medical records were excluded. We analyzed patient demographics, clinical course, kidney transplant function, and postdischarge follow-up to a maximum of 60 days. The study was approved by the Tulane University School of Medicine Institutional Review Board (IRB), and the requirements to obtain informed consent were waived in accordance with 45 CFR 46.116 (d). The IRB did not identify any ethical concerns.

## RESULTS

### Demographics and Baseline Characteristics

Twenty-three patients admitted (11 at one hospital, 12 at the other) with COVID-19 infection were included in this study. The average patient age was 52.9 years, and 95.7% were Black (Table 1). Regarding medical history, 52.2% had a history of diabetes and were obese (body mass index ≥30 kg/m^2^), and 87% had a history of hypertension. The median posttransplant time at the time of hospitalization was 7 years (IQR=10.6)*.* Etiologies of native kidney disease varied and most commonly included diabetes and hypertension. In our population, 78.3% of patients were on a combination of calcineurin inhibitor (tacrolimus/cyclosporine) and antiproliferative agent (mycophenolate mofetil/azathioprine); 73.9% of patients were on maintenance low-dose prednisone. The average baseline serum creatinine (based on values up to 3 months before admission) was 1.8 mg/dL.

**Table 1.** Demographics and Baseline Characteristics, n=23

Variable	Value
Age, years, mean (range)	52.9 (38-80)
Male	16 (69.6)
Black	22 (95.7)
History of hypertension	20 (87.0)
History of diabetes	12 (52.2)
Obesity (BMI >30 kg/m^2^)	12 (52.2)
History of malignancy	3 (13.0)
Time posttransplant to admission, years, median (IQR)	7 (10.6)
Type of transplant	
Kidney alone	18 (78.3)
Kidney and pancreas	3 (13.0)
Kidney and liver	2 (8.7)
Maintenance immunosuppression	
Calcineurin inhibitor plus antiproliferative agent^a^	18 (78.3)
Low-dose prednisone	17 (73.9)
Chronic kidney disease stage	
2	5 (21.7)
3a	8 (34.8)
3b	7 (30.4)
4	3 (13.0)
Baseline serum creatinine, mg/dL, mean (range)	1.8 (1.0-2.8)

^a^Calcineurin inhibitors were either tacrolimus or cyclosporine. Antiproliferative agents were either mycophenolate mofetil or azathioprine.

Note: Data are presented as n (%) unless otherwise indicated.

BMI, body mass index.

### Hospital Course and Outcomes

Presenting symptoms of COVID-19 are shown in Table 2 and included fever, cough, and dyspnea. The most common presenting symptom was fever, present in 52.2% of patients. On average, patients had symptoms for 6.1 days before hospitalization, and 95.7% of patients acquired infection in a community setting. The average length of stay was 4.5 days.

**Table 2.** Presentation, Hospital Course, and Outcomes, n=23

Variable	Value
Symptom duration prior to admission, days, mean (range)	6.1 (1-17)
Presenting symptoms	
Fever	12 (52.2)
Cough	11 (47.8)
Dyspnea	10 (43.5)
Weakness	6 (26.1)
Diarrhea	5 (21.7)
Nausea	5 (21.7)
Myalgias	4 (17.4)
Vomiting	3 (13.0)
Altered mental status	2 (8.7)
Lightheadedness	2 (8.7)
Length of stay, days, mean (range)	4.5 (2-18)
Community-acquired infection	22 (95.7)
Treatment	
Reduction in immunosuppression	23 (100)
Azithromycin	14 (60.9)
Hydroxychloroquine	11 (47.8)
Remdesivir	2 (8.7)
Methylprednisolone pulse	2 (8.7)
Lung infiltrates on initial chest x-ray	15 (65.2)
Venous thromboembolic episode	3 (13.0)
Required mechanical ventilation	9 (39.1)
Mortality of mechanical ventilation subgroup	7 (77.8)
Acute kidney injury	17 (73.9)
Mortality of acute kidney injury subgroup	6 (35.3)
Peak serum creatinine, mg/dL, mean (range)	3.81 (0.8-1.4)
Required renal replacement therapy	6 (26.1)
Received diuretics during admission	5 (21.7)
Received ACE/ARB during admission	2 (8.7)
Serum creatinine at discharge, mg/dL, mean (range)	2.09 (0.9-5.4)
Died from COVID-19 complications	7 (30.4)

Note: Data are presented as n (%) unless otherwise indicated.

ACE, angiotensin-converting enzyme; ARB, angiotensin receptor blocker; COVID-19, coronavirus disease 2019.

All patients were treated initially with a reduction in immunosuppression, most commonly by reducing the dose of antiproliferative agent. Patients received azithromycin (60.9%), hydroxychloroquine (47.8%), remdesivir (8.7%), and intravenous methylprednisolone pulse (8.7%). On initial chest x-ray, 65.2% of the patients had lung infiltrates. Mechanical ventilation was required for 9 (39.1%) patients; 77.8% of patients who required mechanical ventilation died during admission. All patients requiring mechanical ventilation had acute kidney injury (AKI). Venous thromboembolic episodes occurred in 13.0% of patients.

Inflammatory markers were measured at the time of admission, and the average values are presented in Table 3. Interleukin-6 levels were available in 3 patients, with an average value of 16.5 pg/mL. Average white blood cell count upon admission was 6.76 k/μL, while hemoglobin average upon admission was 12.1 g/dL, and platelet count was 214.9 k/μL.

**Table 3.** Laboratory Values on Admission

Test	Mean	Normal Range
C-reactive protein, n=21	61 mg/L	<10 mg/dL
Erythrocyte sedimentation rate, n=8	68 mm/h	Males: 0-22 mm/h
		Females: 0-29 mm/h
Ferritin, n=21	1,609.4 ng/mL	Males: 20-250 ng/mL
		Females: 10-120 ng/mL
Procalcitonin, n=19	1.9855 ng/mL	0.10-0.49 ng/mL
Lactate dehydrogenase, n=21	310.5 U/L	140-280 U/L
Interleukin 6, n=3	16.5 pg/mL	0-16.4 pg/mL
White blood cell count, n=23	6.76 k/μL	5-10 k/μL
Hemoglobin, n=22	12.1 g/dL	Males: 13.8-17.2 g/dL
		Females: 12.1-15.1 g/dL
Platelet count, n=22	214.9 k/μL	140-400 k/μL
Spot urine protein to creatinine ratio, n=6	1.08 g/g	<0.30 g/g

Seventeen patients (73.9%) developed AKI, with an average peak serum creatinine of 3.81 mg/dL. Of patients with AKI, 35.3% (6/17) died. Renal replacement therapy was required for 26.1% of admitted patients. During hospital admission, 21.7% of patients received diuretics. Prior to admission, 39% of patients were on angiotensin-converting enzyme inhibitors or angiotensin receptor blockers, while only 8.7% received them during admission. Urinalysis showed that 77% percent of patients had at least +1 protein, and the average urine protein creatinine ratio during admission was 1.08 g/g. During admission, 30.4% of patients died of COVID-19–related complications. In terms of allograft function post-COVID-19 admission, average serum creatinine was 2.09 mg/dL at discharge compared to an average baseline serum creatinine before admission of 1.8 mg/dL. All patients with baseline stage 4 CKD (13.0%) died.

## DISCUSSION

### COVID-19 Infection Effects on the Kidney

AKI has emerged as a distinct feature of COVID-19 infection, with the incidence in hospitalized patients ranging from 22% to 57% in large US samples.^17-19^ The incidence of severe (stage 3) AKI among patients hospitalized with COVID-19 was higher when compared to COVID-19 negative controls and a historic cohort.^19^ Cheng et al reported a mortality rate of 72% in patients with AKI and COVID-19 compared to 14% mortality in patients without AKI.^20^ Lee et al reported that only 30% of patients with AKI and COVID-19 recovered baseline kidney function and were discharged alive.^21^

Biopsy findings in patients with COVID-19 and AKI have ranged from acute tubular necrosis, to minimal change disease, to collapsing focal segmental glomerulosclerosis.^22-24^ Whether these findings are a consequence of severe systemic disease or direct cytotoxic effects on the kidney is not yet known. However, an autopsy series of 22 patients who died from COVID-19 showed renal tropism of SARS-CoV-2 with preferential targeting of glomerular cells.^25^ These findings are consistent with past evidence of angiotensin-converting enzyme 2 expression in human kidneys, which is known to be the primary receptor for SARS-CoV-2 tissue invasion.^26^

Our results show similar findings of high rates of AKI among COVID-19–infected individuals (73.9%), with a mortality rate of 35.3% among patients with AKI. These findings point to the possibility of an increased risk of AKI among transplant recipients that could contribute to higher death rates. However, no causal inferences can be made from our sample, and whether our high rates of AKI reflect direct kidney targeting by SARS-CoV-2 or a manifestation of systemic disease remains unclear.

### COVID-19 Infection in Patients With Kidney Disease

Nearly 750,000 people in the United States have end-stage renal disease.^27^ The CDC estimates that 15% of US adults have CKD.^28^ Patients with kidney disease often have other comorbidities, putting them at increased risk for more severe COVID-19 infection. An early analysis of COVID-19–infected patients found that patients with CKD represented 9% and 12% of hospitalized non-intensive care unit (ICU) and ICU admissions, respectively.^29^ Furthermore, a meta-analysis of 4 studies that included 1,389 COVID-19–infected patients demonstrated a higher risk of severe infection in CKD patients (odds ratio=3.03, 95% CI 1.09-8.47).^30^ Hemodialysis patients may present atypically (less likely to present with fever, cough, and fatigue compared to nondialysis controls) but have a higher risk of death compared to nondialysis patients with COVID-19.^31,32^

While our sample did not include patients on dialysis at baseline, we observed a mortality rate of 100% in patients with stage 4 CKD, 28.6% in patients with stage 3b CKD, and 12.56% in patients with stage 3a CKD.

### COVID-19 Infection in Kidney Transplant Recipients

Transplant recipients represent a unique patient population in which the role of immunosuppression is unclear. On the one hand, chronic immunosuppression may dampen the hyperinflammatory state that is thought to be a substantial contributor to organ injury in COVID-19 infection. On the other hand, a diminished T cell population may increase the burden of virus, leading to more severe infection. Nevertheless, transplant recipients appear to be at higher risk of mortality, with in-hospital mortality rates ranging from 18% to 32% compared to 14.2% for the general population.^2,3-7^ This increased risk is confirmed by our study, which showed a 30.4% in-hospital mortality.

Similar to patients on hemodialysis, atypical presentations among transplant recipients compared to nontransplant patients have been reported in observational studies, including more patients with gastrointestinal (GI) symptoms (eg, vomiting) and fewer with fever.^16,33^

In a literature review of GI manifestations of COVID-19, Abbasinia et al noted diarrhea and vomiting were the most frequently observed GI symptoms, with rates ranging from 2.0% to 33.7% and 1.0% to 11.1%, respectively.^34^ Rates of diarrhea and vomiting in our sample are close to these observations.

Fever was the most frequently observed presenting symptom in our sample. Huang et al observed a fever rate of 80% in their sample of 41 patients in Wuhan, China, with fever defined as a temperature >38 °C.^35^ Conversely, Richardson et al observed a fever rate of 30.7% in their report on presenting characteristics and outcomes of hospitalized patients with COVID-19 in New York City.^17^ The rate of fever in our sample falls in the middle of these observations, possibly indicating more similarities than differences in the presenting symptoms between transplant and nontransplant patients. Further studies are warranted.

### COVID-19 Infection Effect on Black Patients

Black patients appear to be particularly at higher risk for COVID-19 exposure and severe outcomes compared to White patients. In our study, 95.7% (22/23) of patients were Black. In contrast, from a total of 2,562 actively followed posttransplant patients in both participating centers, only 49% identified as Black. We found an in-hospital mortality rate of 27.2% among Black patients with COVID-19 (6/22 Black patients died) compared to 14.2% nationally. Price-Haywood and colleagues observed similar racial differences in their study of characteristics and clinical outcomes between racial categories.^10^ Black and female patients represented the majority of COVID-19–positive patients*,* and 80% of patients who received critical care or mechanical ventilation were Black. Furthermore, Blacks were overrepresented among all patients who died in the hospital (70.6%) although in-hospital mortality was similar between racial categories after adjustment for differences in socioeconomic and clinical characteristics on admission.^10^ Similar findings were noted in a study published by Gold et al in which 80% of hospitalized patients with COVID-19 in Georgia were Black.^36^

The observed differences in outcomes are likely multifactorial in origin. Price-Haywood et al found a higher prevalence of obesity, diabetes, hypertension, and CKD at baseline in Black patients compared to White patients.^10^ Socioeconomic factors such as type of job or housing density may have prevented social distancing, and racial differences in access to medical care can hinder adequate control of chronic medical conditions.^37^ All of these factors may contribute to the poor outcomes observed in Black patients with COVID-19.

## CONCLUSION

During the initial infection surge in New Orleans, transplant recipients with COVID-19 appeared to present with similar symptoms as nontransplant patients but had an increased risk for AKI and death compared to the general population (ie, in-hospital mortality rate of 14.2%). Most of the patients were Black. Patients sustaining kidney injury and requiring mechanical ventilation had the worst outcomes. Kidney function did not return to baseline at discharge in patients sustaining AKI. Specific treatment regimens for transplant recipients remain to be determined.

## Notes

*This article meets the Accreditation Council for Graduate Medical Education and the American Board of Medical Specialties Maintenance of Certification competencies for Patient Care, Medical Knowledge, and Practice-Based Learning and Improvement.*
